# Encapsulated Cell Dynamics in Droplet Microfluidic Devices with Sheath Flow

**DOI:** 10.3390/mi12070839

**Published:** 2021-07-19

**Authors:** Peter E. Beshay, Ali M. Ibrahim, Stefanie S. Jeffrey, Roger T. Howe, Yasser H. Anis

**Affiliations:** 1 Mechanical Design and Production Department, Faculty of Engineering, Cairo University, Giza 12613, Egypt; beshay.6@buckeyemail.osu.edu (P.E.B.); ali.ibrahim2@mail.mcgill.ca (A.M.I.); 2 Department of Surgery, Stanford University School of Medicine, Stanford, CA 94305, USA; ssj@stanford.edu; 3 Department of Electrical Engineering, Stanford University, Stanford, CA 94305, USA; rthowe@stanford.edu

**Keywords:** microfluidics, droplets, emulsions, cell biomechanics, sheath flow

## Abstract

In this paper we study the dynamics of single cells encapsulated in water-in-oil emulsions in a microchannel. The flow field of a microfluidic channel is coupled to the internal flow field of a droplet through viscous traction at the interface, resulting in a rotational flow field inside the droplet. An encapsulated single cell being subjected to this flow field responds by undergoing multiple orbits, spins, and deformations that depend on its physical properties. Monitoring the cell dynamics, using a high-speed camera, can lead to the development of new label-free methods for the detection of rare cells, based on their biomechanical properties. A sheath flow microchannel was proposed to strengthen the rotational flow field inside droplets flowing in Poiseuille flow conditions. A numerical model was developed to investigate the effect of various parameters on the rotational flow field inside a droplet. The multi-phase flow model required the tracking of the fluid–fluid interface, which deforms over time due to the applied shear stresses. Experiments confirmed the significant effect of the sheath flow rate on the cell dynamics, where the speed of cell orbiting was doubled. Doubling the cell speed can double the amount of extracted biomechanical information from the encapsulated cell, while it remains within the field of view of the camera used.

## 1. Introduction

Emulsion-based microfluidics has various important applications in chemistry and biology, ranging from drug discovery to biomolecule synthesis [1,2,3,4]. Emulsions are droplets of one fluid that are dispersed in another fluid, usually oil-in-water or water-in-oil. They provide the advantage of using very small volumes of reagents and samples, which enable different biological and chemical processes to be both fast and inexpensive.

Droplet microfluidics has recently emerged as a powerful technology for single-cell manipulation [5,6,7,8]. Aqueous droplets in oil provide small isolated volumes, engineered environments, controllable vessels, and reduced shear stress levels on the encapsulated cells. The potential of droplet microfluidics has also been recently demonstrated for the label-free detection of rare cells based on their biomechanical properties [9]. Different techniques have been used to identify or sort cells within a heterogeneous sample. Affinity-labeled techniques commonly use antibodies against the cell surface protein and have the advantage of high specificity and multiplexing capability [10,11], while label-free techniques are based on the detection of physical properties such as cell size, deformability, compressibility, shape, density, surface properties, electrical polarizability, magnetic susceptibility, and refractive index [12,13,14]. Label-free cell identification techniques have the advantages of high sorting throughput, requiring no immunostaining, antibody labeling, or expensive chemical reagents [15]. Microfluidics label-free cell sorting mechanisms can include microfilters [16], pinched flow fractionation (PFF) [17], deterministic lateral displacement (DLD) [18], inertial sorting [19], hydrodynamic filtration [20], magnetophoresis [21], dielectrophoresis [22], and acoustophoresis [23].

Emulsion biomechanics is another label-free biomechanics technique for cell detection, where single cells are encapsulated inside water-in-oil emulsions and studied under various microfluidic conditions [9]. Emulsion biomechanics techniques can include (1) direct deformation and (2) accelerating flow. In direct deformation, droplets are compressed into an ellipsoidal shape and then allowed to relax back to spherical. Upon relaxation, a flow field results inside the droplet causing the encapsulated cell to respond mechanically through measurable deformation. In accelerating flow, the flow field of a microfluidic channel is coupled to the internal flow field of the droplet through viscous traction at the interface resulting in a rotational flow field inside the droplet. An encapsulated single cell being subjected to this flow field responds by undergoing multiple orbits, spins, and deformations that depend on its physical properties. The rotational flow field (vortex) inside the droplet is the result of the coupling between the external flow field—outside the droplet—and the internal flow field—inside the droplet. The flow fields are very complex and depend on several factors, including the viscosity μ of the dispersed and continuous phases and the capillary number. In addition, the position and number of vortices inside a droplet depends on the size and the shape of the droplet, as well as the interfacial tension.

In this paper, we study the dynamics of single cells encapsulated in water-in-oil emulsions in a microchannel. Monitoring the cell dynamics, using a high-speed camera, can lead to the development of new label-free methods for the detection of rare cells, based on their biomechanical properties. A sheath flow microchannel is proposed to strengthen the rotational flow field inside droplets flowing in Poiseuille flow conditions. A numerical model is developed to investigate the effect of various parameters, including the sheath flow rate, on the rotational flow field inside a droplet. The effect of the sheath flow rate on the cell dynamics was demonstrated experimentally.

## 2. Rotational Flow Field Inside a Droplet

Emulsions are considered as controlled microenvironments, where the physical and chemical parameters of this environment control the behavior of the encapsulated cells. They are usually generated using T-junction, cross-junctions, or co-flow microchannels [24,25]. To maintain the stabilization of the droplets, chemical agents called surfactants are used. The fluidic conditions inside the droplet differ from outside in two main aspects: (1) the existence of a rotational flow field (vortex) inside the droplet and (2) the pressure difference between the inside of the droplet and the outside fluid (△p) is positive [26]. △p can be expressed using the following Young–Laplace equation:

(1) $$△p=\frac{2\gamma }{R_{e}},$$

where $R_{e}$ is the radius of the spherical droplet, and γ is the interfacial tension between the droplet (dispersed phase) and the surrounding fluid (continuous phase). The surfactant type affects γ, thus also affecting the shape and size of the droplet, in addition to the pressure inside.

When a droplet is disturbed from its stationary position, a rotational flow field emerges inside the droplet, which causes encapsulated cells to spin. Without this perturbation, no flow field will form inside the droplet and the encapsulated cell will remain stationary. Means to perturb a droplet from its stationary position include forcing the droplet to vertically drop or rise inside a microchannel, where both the density difference between the continuous and dispersed phases and the viscous stresses along the interfacial surface of the droplet will cause external and internal flow fields to emerge [27]. Electrowetting has been used to disturb droplets by using electric fields that introduce electrical forces that act as body forces onto the droplets, controlling the flow field inside [28]. The presence of the interfacial tension gradient causes the Marangoni effect, where a flow gradient forms along the surface, hence, causing a rotational flow field [29]. The interfacial tension gradient can result from a temperature gradient along the surface of the droplet or from the presence of a surfactant [30]. This effect usually interferes with other rotational flow fields, resulting from viscous stresses inside the droplet, complicating the flow field and making it hard to model.

Another source of droplet disturbance is the application of an external flow field, which causes the droplets to deform, generating strong rotational flow fields inside. The field is a result of the viscous traction forces generated during the relaxation of the droplet, which depends on the capillary number $C_{a}$, as was first introduced by Taylor [31]. The capillary number $C_{a}$ represents the ratio between the viscous forces and the interfacial tension of the droplet, represented as:

(2) $$C_{a}=\frac{\mu_{o}\dot{\tau }R_{e}}{\gamma }\;,$$

where $\mu_{o}$ is the viscosity of the external fluid, $\dot{\tau }$ is the shear rate of the external flow field, $R_{e}$ is the radius of the spherical droplet, and γ is the interfacial tension of the fluid–fluid interface.

While the viscous forces of the external fluid work on deforming the droplet, the interfacial tension acts as a spring that opposes this deformation such as to restore the droplet back to its original shape. Droplets with low $C_{a}$ values indicate that the interfacial tension is the most dominant parameter; hence, the droplet becomes more resistant to deformation. Consequently, the rotational flow field inside the droplet becomes weaker. On the other hand, droplets with high $C_{a}$ values tend to deform under the effect of external flow fields, thus, stronger internal flow fields are generated.

### 2.1. Analytical Model of the Droplet Flow Field

Analytical expressions of internal and external flow fields are driven under very restrictive assumptions, which include that: (a) droplets are small, (b) droplets remain spherical during the flow, (c) there is a condition of no-slip at the droplet interface, and (d) the tangential stress across the interface is continuous [31,32]. The extensional flow and the simple shear flow can be expressed in the spherical coordinates (*r*,ϕ) along a streamline *k* inside a droplet of radius $R_{e}$ as:

(3) $$\frac{r^{3}}{R_{e}^{3}}\left(1-\frac{r^{2}}{R_{e}^{2}}\right)=\frac{k}{{\left(sin2\phi \right)}^{\frac{3}{2}}}\;,\mathrm{and}$$

(4) $${\left[\frac{r^{2}}{R_{e}^{2}}cos2\phi +\left(\lambda +1\right)\right]}^{3}{\left[\frac{r^{2}}{R_{e}^{2}}-1\right]}^{2}=k\;,$$

where λ is the ratio between the dispersed phase and the continuous phase viscosities.

### 2.2. Numerical Model of the Droplet Flow Field

Taylor and other researchers worked on experimentally verifying their analytical models. However, doing so was difficult due to the complex effect of the surfactants. Taylor proposed a device called the four-roll mill that was capable of inducing extensional as well as simple shear flow [31,32,33]. This device was unstable, as it was difficult to maintain the droplet at the center of the apparatus. Similarly, Lee et al. introduced a microfluidic chip that consists of four inlets and outlets [34]. The fluid flow rate inside each channel determined the type of the external flow field applied to the centered droplet. As a result of its symmetric design, it was possible to obtain a pure rotational external flow field.

The numerical model in this paper is developed based on Taylor’s concept to further investigate the effect of various parameters on the rotational flow field inside a water-in-oil emulsion droplet. In contrast to single-phase flow problems, the modeling of multi-phase flow problems is complex due to the need to solve a time-dependent problem. Furthermore, modeling multi-phase flow requires the tracking of the fluid–fluid interface, which deforms over time or, more complexly, breaks up. The complexity of the boundary conditions and the interfacial tension gradient, which causes the Marangoni effect, also increases the overall complexity of the model.

## 3. Modeling

The model was constructed as in [33,34], where the droplets are kept stagnant while being introduced to a Poiseuille flow. Our approach to solving this multi-phase model begins by solving Navier–Stokes momentum equations to get the pressure and velocity fields. These fields are then applied to the interface as boundary conditions in the form of shear stresses. The fluid–fluid interface is tracked while deforming over time due to the applied shear stresses. Finally, the distribution of phase in each fluid is evaluated and the process is repeated until convergence is achieved, as in [35,36]. The effect of the generated droplet flow fields on the dynamics of a cell suspended inside of the droplet is investigated. It is worth mentioning that the Reynolds numbers for the multiple phases are low; thus, laminar flow models are used.

### 3.1. Model Definitions

**Figure 1:**
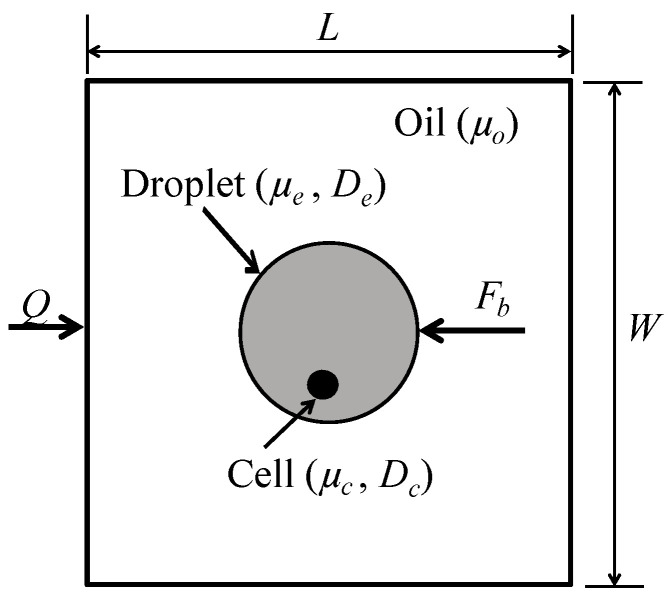
Modeling of rotational flow of a single cell is encapsulated inside an droplet.

A continuous-phase fluid of viscosity $\mu_{o}$ enters a channel of width *W* and length *L* with a flow rate $Q_{co}$, as illustrated in Figure 1. In this model, the water-in-oil emulsion droplet of diameter $D_{e}$ and viscosity $\mu_{e}$ is kept stagnant by means of the body force $F_{b}$; hence, the droplet velocity is kept at zero. The difference between the continuous phase velocity and the droplet velocity generates viscous forces on the droplet. These viscous forces, coupled with the interfacial tension of the fluid–fluid interface γ, generate the rotational flow field. The droplet encapsulates a suspended cell, which is modeled as a fluid droplet of diameter $D_{c}$ and viscosity $\mu_{c}$, as shown in Figure 1.

Computational Fluid Dynamics (CFD) simulations were performed numerically by solving the following non-slip, incompressible flow Navier–Stokes equations using a finite element package (COMSOL Multiphysics^®^, Stockholm, Sweden):

(5) $$\rho_{e}\frac{\partial u_{e}}{\partial t}=-\nabla p_{e}+\mu_{e}\nabla^{2}u_{e}+\rho_{e}g+F_{st}+F_{b}\;,$$

(6) $$\nabla \cdot u_{e}=0\;,$$

where $\rho_{e}$ is the density of the droplet (kg/m${}^{3}$), $u_{e}$ is the droplet velocity field (m/s), *t* is the time (s), $p_{e}$ is the pressure field on droplet (Pa), $\mu_{e}$ is the viscosity of the droplet (Pa·s), *g* is the gravitational acceleration (m/s${}^{2}$), $F_{st}$ is the surface force resulting from the interfacial tension (N), and $F_{b}$ is the body force acting on the droplet (N).

Simulation of cell movement inside the droplet is performed numerically by solving the following Newtonian equation of motion for the cell:

(7) $$\frac{d\left(m_{c}v_{c}\right)}{dt}=F_{d}\;,$$

where $m_{c}$ is the mass of the cell (kg), and $v_{c}$ is the velocity of the cell (m/s). The drag force ($F_{d}$) acting on the cell (N) is represented by:

(8) $$F_{d}=\frac{3\mu_{c}C_{D}Re_{c}}{4\rho_{c}D_{c}^{2}}m_{c}\left(u_{e}-v_{c}\right)\;,$$

where $\mu_{c}$ is the viscosity of the cell (Pa·s), $\rho_{c}$ is the density of the cell (kg/m${}^{3}$), $D_{c}$ is cell diameter (m), $u_{e}$ is the velocity field of the droplet obtained from solving (5) and (6), and $C_{D}$ is the drag coefficient, represented by:

(9) $$C_{D}=\frac{8}{Re_{c}}\left(\frac{2+3(\mu_{c}/\mu_{e})}{1+(\mu_{c}/\mu_{e})}\right)\;,$$

where $Re_{c}$ is the Reynolds number of the cell:

(10) $$Re_{c}=\frac{\rho_{c}\left|u_{e}-v_{c}\right|D_{c}}{\mu_{e}}.$$

### 3.2. Numerical Simulations: Flow Field

The governing equations were solved for the case where the cell diameter $D_{c}=10$ µm, cell viscosity $\mu_{c}=100$ mPa·s, droplet diameter $D_{e}=50$ µm, droplet viscosity $\mu_{e}=1$ mPa·s, flow rate $Q_{co}=1$ µL/min, and the fluid viscosity $\mu_{o}=150$ mPa·s. The shear modulus of the membrane of the cell $\gamma_{c}$ was set to 1 mN/m. The interfacial tension γ=5 mN/m is a typical value for water-in-oil emulsions [9]. The body force per cell volume acting on the droplet was determined by trial and error as 3.25 nN/m${}^{3}$ (i.e., $F_{b}=3.25\;\times$ Volume). The dimensions of the channel were 150 × 150 × 50 µm for the length (*L*), width (*W*), and thickness (*T*), respectively.

The equations were solved using PARDISO time dependent solver with nested dissection multithreaded preordering algorithm. We used Quad elements to create the mesh of the external fluid with a maximum element size of 6.75 µm. The droplet was meshed using triangular elements with a maximum element size of 0.5 µm. The complete mesh consisted of 6628 domain elements and 408 boundary elements. Channel walls had no-slip boundary conditions. The inlet of the external fluid had an entrance length of 1200 µm to ensure that the flow is fully developed. More details about the numerical model are available as Supplementary Material.

**Figure 2:**
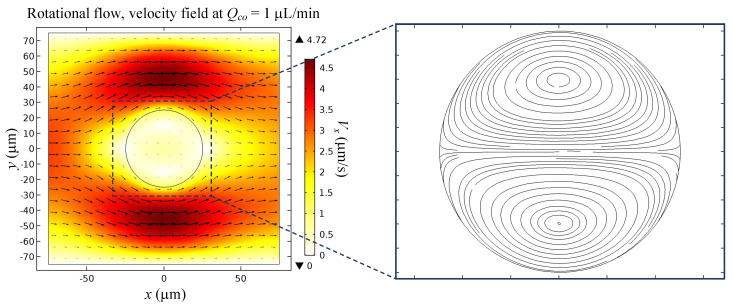
External flow field inside and outside a droplet at $Q_{co}$ = 1 µL/min due to the 4.5 mm/s velocity difference between the droplet and the external fluid.

CFD simulations resulted in a 4.5 mm/s velocity difference between the droplet and the external fluid, as depicted in Figure 2, which also shows the continuous phase fluid streamlines flow around the droplet. This disturbance causes the generation of a viscous force that opposes the interfacial tension to generate a rotational flow field inside the droplet. The velocity field streamlines in Figure 2 indicate that the two vortices inside the droplet are symmetrical. Furthermore, there are two stagnant points, which can be observed at the coordinates (0, 15) and (0, −15).

### 3.3. Numerical Simulations: Cell Dynamics

**Figure 3:**
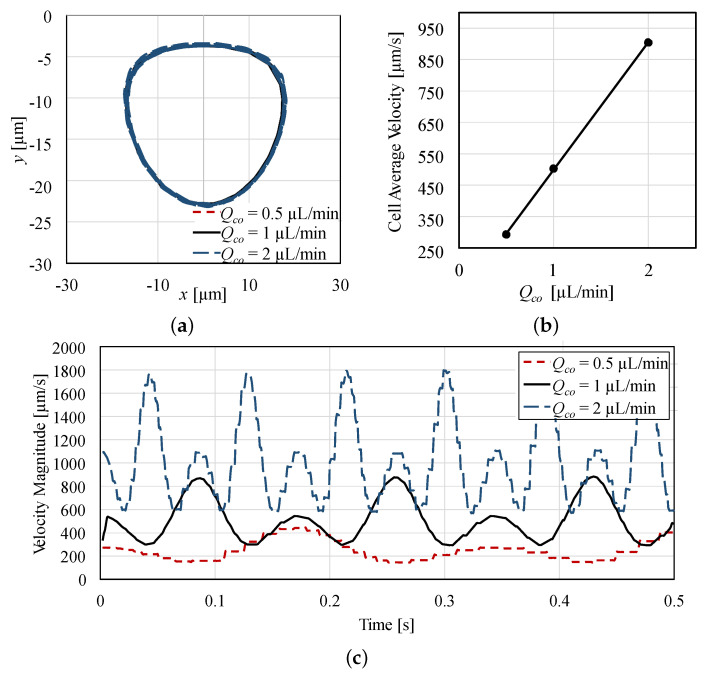
Effect of flow rate on encapsulated cell dynamics: (**a**) cell trajectory, (**b**) cell average velocity, and (**c**) cell velocity profile.

CFD simulations resulted in the cell trajectory presented in Figure 3a, at three different flow rates: $Q_{co}=$ 0.5, 1, and 2 µL/min. Body forces $F_{b}=$ 1.625, 3.25, and 6.5 nN/m${}^{3}$ were required to hold the droplet in place. As a result, the velocity difference between the droplet and the surrounding fluid were calculated as 2.25, 4.5, and 9 mm/s, respectively.

The initial cell position was arbitrarily selected at the position (x=0, y=−4). The cell continued to move repeatedly in an elliptical orbit, while also spinning around its center. It is worth mentioning that the streamline at which the cell orbits depends on the cell’s initial position. If the cell initial position was at the stagnation point, it will continue to spin around its center while maintaining its position relative to the droplet.

Figure 3a also shows that the flow rate $Q_{co}$ had a negligible effect on the cell trajectory, which means that increasing the flow rate does not affect the shape of the streamlines inside the droplet. On the other hand, $Q_{co}$ seems to have a highly significant effect on the cell velocity. As depicted in Figure 3b, cells had average velocities of 293, 503, 905 µm/s at the flow rates $Q_{co}=$ 0.5, 1, 2 µL/min, respectively. There was an almost linear relation between the increase in the flow rate and the increase in average cell velocity. With the droplet held stationary, the increase in the velocity of the continuous phase was translated into an increase in the relative velocity between the dispersed and continuous phases. This increased the strength of the rotational flow field inside the droplet, which in turn increased the velocity of the encapsulated cell.

Figure 3c plots the translational velocity ($|v_{c}|$) of the encapsulated cell with time, which fluctuates with the changing cell position in the elliptical orbit. It shows that the velocity of the cell is higher when it is orbiting farther from the droplet center than nearer. Figure 3c also shows that increasing $Q_{co}$ leads to an increase in both the cell orbiting frequency and the velocity amplitude. This result is very important as it will later be discussed how strong rotational flow fields can be used to collect more cell-biomechanics information in less time compared to at weaker flow fields.

## 4. Sheath Flow for Increased Encapsulated Cell Velocity

**Figure 4:**
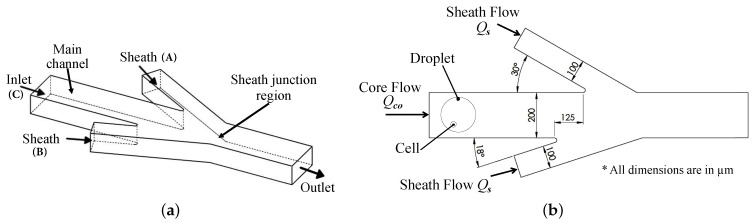
Schematic of the sheath flow microchannel, consisting of three inlet ports: one for core flow and two for sheath flow: (**a**) microchannel inlets and outlet, and (**b**) microchannel dimensions.

Sheath flow microchannels were proposed to strengthen the rotational flow field inside droplets flowing in Poiseuille flow conditions. Figure 4 shows a schematic of the rotational flow-inducing sheath-flow channels. Two sheath flow microchannels, (A) and (B), were designed, inclined at 18${}^{\circ }$ and 30${}^{\circ }$ angles to the horizontal main channel (C). The widths of the main and sheath channels are selected as 200 µm, and 100 µm, respectively. A 125 µm horizontal offset was created between the two sheath channels. The asymmetry of the sheath channels (A) and (B), in addition to the horizontal offset, generate a moment on the flowing droplet. In turn, this moment strengthens the rotational flow field inside the droplet. Furthermore, the vertical components of the sheath flow from both (A) and (B) work on deforming the droplet as it passes through the sheath junction region shown in Figure 4a.

After passing the sheath region, a strong rotational flow field is generated inside this droplet, caused by the viscous traction forces that occur during the relaxation of the droplet, which depends on the droplet’s capillary number $C_{a}$. The sheath flow rate should be selected within a reasonable range so that the deformation of the droplet does not affect the encapsulated cell viability.

### 4.1. Numerical Simulations

Computational Fluid Dynamics (CFD) simulations were conducted to numerically study the parameters that affect the rotational flow field inside a droplet that flows in a sheath flow microchannel. The studied parameters include the core flow rate $Q_{co}$, the sheath flow rate $Q_{s}$, the generated droplet diameter $D_{e}$, the cell diameter $D_{c}$, the continuous phase viscosity $\mu_{f}$, and the droplet viscosity $\mu_{e}$. The percent increase in the velocity of the encapsulated cell after passing through the sheath region $I_{v}$ was selected as the parameter that represents the effect of the sheath region on the flow field inside the droplet:

(11) $$I_{v}=\frac{v_{cas}-v_{cbs}}{v_{cbs}}\times 100\;,$$

where $v_{cbs}$ is the average velocity of the cell before introduction to sheath flow, while $v_{cas}$ is the average velocity of the cell after introduction to sheath flow. The sheath flow rate in both sheath channels $Q_{s}$ (µL/min) is assumed to be equal, as shown in Figure 4b.

The CFD simulations of the droplet movement were performed by solving the non-slip, incompressible flow Navier–Stokes Equations (5) and (6). Simulation of cell movement inside the droplet was performed numerically as described in Section 3.3. The simulations were conducted to screen all parameters and determine the ones that had the most significant effect on the percent increase in cell velocity $I_{v}$, which also represents the increase in the rotational flow field. Each parameter had its value varied between a lower and a higher value, as presented in Table 1.

$Q_{s}$ was selected between 0.5 and 2 µL/min. Higher sheath flow rates are not permissible as they may affect the viability of the cells. $D_{e}$ was varied between 70 and 190 µm. It worth mentioning that $D_{e}$ is not directly controlled but depends on the flow rates at the emulsification junction [37]. $D_{c}$ was varied between 7 µm, a typical value of RBCs, and 25 µm, which corresponds to larger WBCs. $\mu_{f}$ was varied between 50 mPa·s as in light mineral oil and 200 mPa·s as in heavier mineral oils. The interfacial tension γ was kept at 5 mN/m. $\mu_{e}$ was varied between 1 mPa·s as in phosphate-buffered saline (PBS) and 5 mPa·s as in typical cell growth medium. Finally, the cell viscosity $\mu_{c}$ was kept constant at 450 mPa·s as in circulating tumor cells [9].

The equations were solved using the PARDISO time-dependent solver with nested dissection multithreaded preordering algorithm. We used triangular elements to create the mesh of the external fluid and the droplet with maximum element size of 15 µm, and 1 µm, respectively. The complete mesh consisted of 14,553 domain elements and 797 boundary elements. Channel walls had no-slip boundary condition. The inlet of the external fluid had an entrance length of 4200 µm to ensure that the flow is fully developed.

### 4.2. Numerical Simulation Results

**Figure 5:**
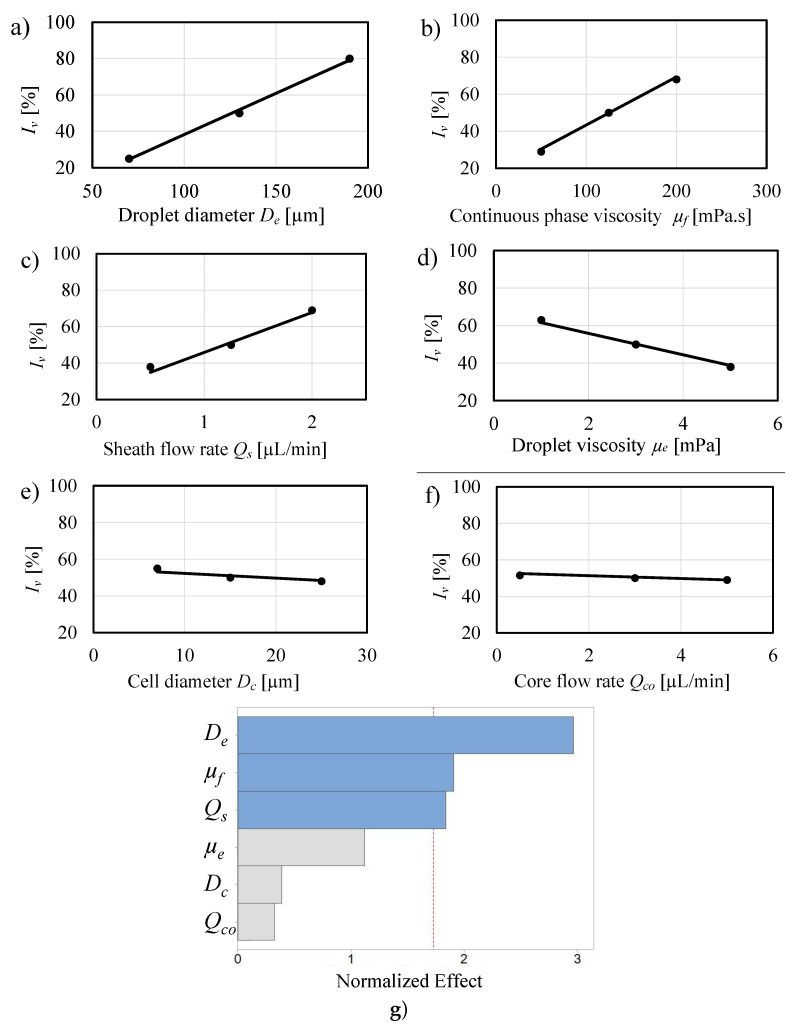
Effect of various microfluidic and cell related parameters on the rotational flow field: (**a**) Effect of $D_{e}$ on $I_{v}$, (**b**) Effect of $\mu_{f}$ on $I_{v}$, (**c**) Effect of $Q_{s}$ on $I_{v}$, (**d**) Effect of $\mu_{e}$ on $I_{v}$, (**e**) Effect of $D_{c}$ on $I_{v}$, (**f**) Effect of $Q_{co}$ on $I_{v}$, (**g**) Pareto chart with parameters sorted in the order of significance.

Figure 5a–f plot the individual effect of each parameter on the rotational flow field due to passing through the sheath flow region, represented by the increase in the rotational flow field $I_{v}$ (see Equation (11)). The figure reveals that $D_{e}$, $\mu_{f}$, and $Q_{s}$ have the largest slopes, hence the greatest effects on $I_{v}$. Figure 5a indicates that as $D_{e}$ increased from 70 µm to 190 µm, $I_{v}$ increased from 25% to 80%. That is due to the increase of the viscous stress effect on the droplet with the increase of $D_{e}$. Figure 5b,c show a significant increase in $I_{v}$ with the increase in $\mu_{f}$ and $Q_{s}$. An increase in $\mu_{f}$ and $Q_{s}$ causes the viscous forces acting on the droplet to also increase, which leads to an increase in the viscous stresses, thus an increase in both the rotational flow field inside the droplet and $I_{v}$.

Figure 5d–f show the effect of some less significant parameters on the flow field of the droplet. As depicted in Figure 5d, $I_{v}$ decreased from 61% to 38% as $\mu_{e}$ increased from 1 mPa·s to 5 mPa·s, in an inverse correlation. This correlation can be explained by the fact that the droplet fluid acts as a viscous damper for the encapsulated cell. As $\mu_{e}$ increases, the droplet damping coefficient increases, leading to the decrease in the velocity of the cell. Figure 5e,f show that the correlation lines are nearly horizontal, indicating that the effects of $D_{c}$, and $Q_{co}$ on the rotational flow field are almost negligible, and also indicate that the sheath flow microchannel can achieve an acceptable velocity increase regardless of the size of the encapsulated cell.

Figure 5g is a Pareto chart that presents the effects of all seven parameters on the flow field. The bars’ lengths resemble the standardized effects, also known as the *T*-value. For a linear relationship, the *T*-value is calculated by the following formula [38]:

(12) $$T-value=\frac{\bar{X_{1}}-\bar{X_{2}}}{\sigma /\sqrt{n}}\;,$$

where $\bar{X_{1}}$ and $\bar{X_{2}}$ are the means of the response, $I_{v}$, at the factor’s higher and lower values, respectively, σ is the standard deviation of the response, and *n* is the number of readings.

Figure 5g confirms that $D_{e}$ has the biggest effect, followed by the continuous phase fluid viscosity $\mu_{f}$ and sheath flow rate $Q_{s}$. The vertical dashed line in Figure 5g represents the significance threshold at a 90% confidence level. The value of this threshold is calculated based on Pseudo-standard error (PSE) using Minitab statistical analysis software (Minitab Inc., State College, PA, USA). According to the threshold, $\mu_{e}$, $D_{c}$, and $Q_{co}$ are confirmed as statistically insignificant parameters due to their negligible effect on the viscous stresses or the interfacial tension of the emulsion. Only $Q_{s}$ and $\mu_{f}$ have direct effects on the viscous stresses.

## 5. Experimental Cell Dynamics in Emulsions

Here, experiments were performed to investigate the dynamics of an encapsulated cell inside a water-in-oil emulsion, which reflects the rotational flow field inside the droplet. Furthermore, the effects of the sheath flow microchannels, the droplet diameter $D_{e}$, and the core fluid flow rate $C_{co}$ on the cell dynamics are also investigated.

### 5.1. Experimental Setup

**Figure 6:**
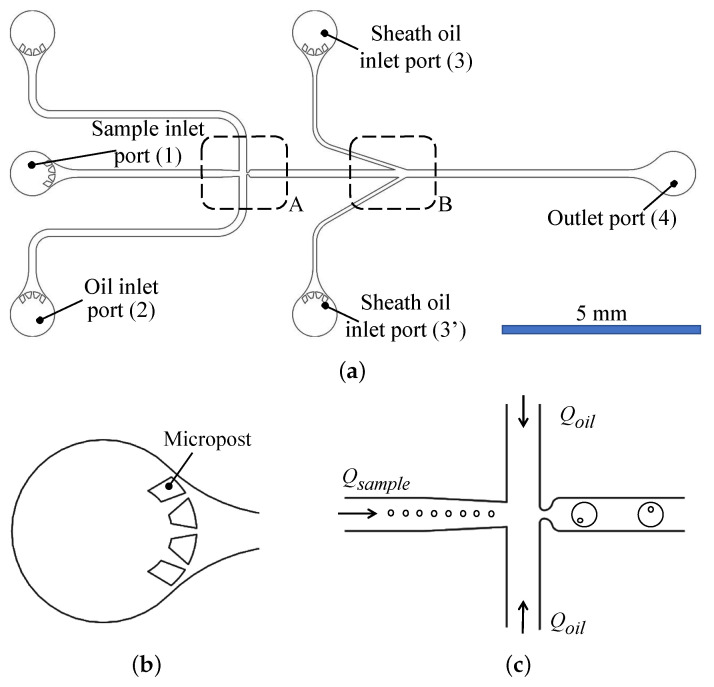
Sheath flow microfluidic chip: (**a**) The chip contains five inlet ports, one for the sample, two for the continuous oil phase, and two for sheath flow. (**b**) Micropost filtration. (**c**) Cross junction emulsification stage.

Figure 6a shows the microfluidic chip design drawn to scale. The chip has six ports: one sample inlet port (1), two core oil inlet ports (2), two sheath oil inlet ports (3 & 3’), and one outlet port (4). Each inlet port contains a set of microposts that filter the fluids before entering the channel (see Figure 6b). This helps prevent unwanted particles from clogging the channels.

Cell growth medium, with suspended cells, is used as the dispersed phase and is flown into the chip through the inlet port (1). At the emulsification region (A), the medium meets the emulsification oil, used as the continuous phase, supplied through the inlet ports (2). In region (A), droplets of cell growth medium are generated inside the emulsifying oil fluid, as depicted in Figure 6c.

The shape and size of the cross junction, surfactant concentration, in addition to the flow rates of the continuous and dispersed phases, determine the size of the generated droplet [37,39]. Here, all cross-junction channels are 200 µm wide and 60 µm deep. After the droplets are generated, they are flown towards the sheath flow stage (B), which consists of two sheath flow microchannels (3) and (3’), inclined at 18${}^{\circ }$ and 30${}^{\circ }$ to the horizontal main channel, as shown in Figure 4b. There is a 125 µm horizontal offset between channels (3) and (3’). The widths of the main and sheath channels are 200 µm and 100 µm, respectively. The microfluidic circuit is 60 µm deep.

Dual-syringe pumps (Pump 11 Pico Plus Elite, Harvard Apparatus, Cambridge, MA, USA) were used to supply the input fluids to the microfluidic device inlet ports through 22-gauge steel pins and Tygon tubing (Instech Laboratories, Plymouth Meeting, PA, USA). Mineral oil (Sigma-Aldrich, St. Louis, MO, USA) was used as the continuous phase. Span80 surfactant (Sigma-Aldrich, St. Louis, MO, USA) was used at a 0.75% [wt/wt] percentage to stabilize the droplets, prevent coalescing, and reduce surface tension. For the sheath flow, we used a mineral oil-based ferrofluid that was prepared as described by [40]. Ferrofluid was selected such that the contrast between the sheath and the dispersed phase is visible during the imaging of droplets. The image capture system included a high-speed sCMOS camera (Andor^TM^ Zyla 4.2, South Windsor, CT, USA), connected to an inverted fluorescent microscope (Eclipse Ti-E, Nikon, Tokyo, Japan) with a 10× objective lens (CFI ACHRO ADL 10× NA .25 WD 5.2 mm Ph1, Nikon, Tokyo, Japan).

### 5.2. Chip Fabrication

The chips were fabricated from Polydimethylsiloxane (PDMS) using soft lithography techniques [41]. The master mold was made on a silicon wafer using SU-8 photoresist (MicroChem, Newton, MA, USA) by standard photolithography techniques. After the preparation of the master mold, uncured Sylgard 184 PDMS and curing agent (Dow Corning, Midland, MI, USA), mixed at a 10:1 ratio, were applied to the mold. The mixture was then cured by baking in an oven at 60${}^{\circ }$ C for 2 hours. The cured PDMS was then peeled from the PDMS, where the port holes were punched. The chips were cut and bonded to cover-slip glass using air-plasma activation in a plasma cleaner (Harrick Plasma PDS-32G, Ithaca, NY, USA).

### 5.3. Cell Preparation

We used the MCF-10A cell line (CRL-10317, ATCC, Manassas, VA, USA), which is a non-tumorigenic epithelial cell line. The cell line was cultured in DMEM/F12 cell culture medium (Dulbecco’s Modified Eagle Medium: Nutrient Mixture F-12), supplemented with 10% Fetal Bovine Serum (FBS) and 1% penicillin-streptomycin (Invitrogen, Grand Island, NY, USA). Cells were kept in a 5% CO${}_{2}$ incubator (VWR, Radnor, PA, USA) at 37${}^{\circ }$. For the experiments, cells were centrifuged twice for 5 min at 800 rpm and suspended in PBS (Phosphate-Buffered Saline) with 0.1% Pluronic F68 (BASF, Florham Park, NJ, USA), and 10% Ficoll PM400 (Sigma Aldrich, St. Louis, MO, USA) at concentrations between $10^{5}$ and $10^{6}$ cells/mL. Pluronic F68 was added to prevent biofouling, while Ficoll was added to increase the buoyancy of the cell suspension.

### 5.4. Experiments

Experiments were conducted at core flow rates $Q_{co}$, varied between 0.35 µL/min and 4.9 µL/min, and droplets of diameters $D_{e}$ that ranged from 70 µm to 197 µm. The sheath flow rate $Q_{s}$ was kept constant at 1 µL/min for all experiments. MCF-10A cell lines have cell diameters $D_{c}$ ranging between 14 and 18 µm. The MCF-10A cell line is naturally fluorescent; thus, image sequences of MCF-10A were performed using fluorescence microscopy, in addition to dim bright field illumination. The frame rates of the imaged sequences ranged between 278 and 933 fps.

**Figure 7:**
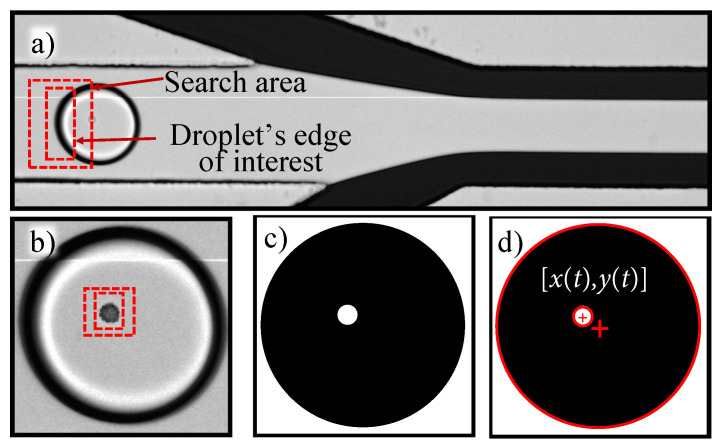
Image sequence processing approach: (**a**) droplet edge is detected and tracked within a defined search area, (**b**) cell is detected and tracked within a defined search area, (**c**) image sequence is converted to black stagnant droplet containing a white moving cell, (**d**) cell position is recorded at every time frame using circular Hough transform.

Raw image sequence data were recorded for all experimental runs using the high-speed camera. These data was then processed and features were extracted to calculate the cell displacement and velocity using the following procedure: (1) Droplets and encapsulated cells were detected and tracked using a contour tracking algorithm to identify and track the droplet’s edge within the defined search region in a frame by frame basis (see Figure 7a). (2) A similar contour tracking algorithm was used for the identification and tracking of the cells (see Figure 7b). (3) The resulting image sequences were black droplets containing white-colored cells, whose centroids were tracked using the MATLAB circular Hough transform algorithm (MathWorks^®^, Natick, MA, USA) [42] (see Figure 7c,d). The position of cell centroids with respect to droplet center was then calculated to obtain the position of the cell. (4) The cell position data were numerically differentiated to obtain the cell velocity.

### 5.5. Results

**Figure 8:**
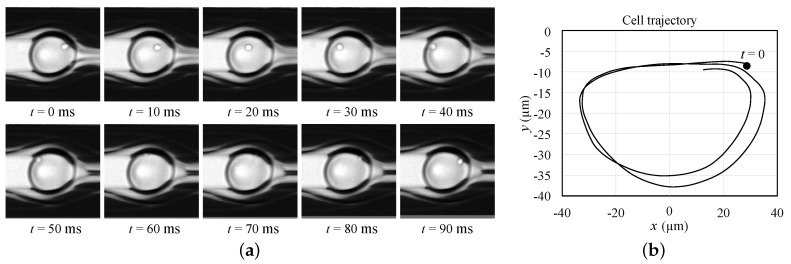
Experimental cell behavior: (**a**) time lapse still images showing MCF-10A cell position at different time frames, (**b**) cell spiral trajectory.

As discussed in Section 2, viscous stresses acting on a droplet generate a rotational flow field inside the droplet, causing an encapsulated cell to rotate in an elliptical orbit. This behavior was confirmed experimentally, as shown in Figure 8a, which shows the cell’s movement over time. The cell depicted in Figure 8a is an MCF-10A cell encapsulated by a droplet of diameter $D_{e}$ = 95 µm, at core flow rate $Q_{co}$ = 1.9 µL/min and sheath flow rate $Q_{s}$ = 1 µL/min. The capture frame rate was 406.9 fps. Figure 8b shows how the cell spins in an elliptical orbit.

#### 5.5.1. Cell Dynamics in Sheath Flow

**Figure 9:**
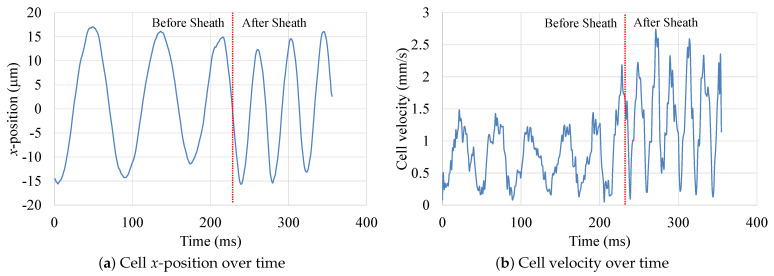
Effect of sheath flow on MCF-10A cell velocity. Cell mean velocity increased by 90%.

The sheath flow microchannel was used to increase the rotational flow field strength inside the droplet, hence increasing the translational velocity of the encapsulated cell. Figure 9 shows the *x*-direction movement of an MCF-10A cell encapsulated inside a droplet of $D_{e}$ = 74 µm diameter, at a core fluid flow rate $Q_{co}=$2.05 µL/min, while keeping the sheath flow rate $Q_{s}$ at 1 µL/min. The capture frame rate was 933 fps. The time rate of change in cell’s *x*-position corresponds to the cell’s speed of rotation Ω (rev/s).

Figure 9a shows how Ω increased after the droplet was subjected to the sheath flow (at *t* = 230 ms). The speed of rotation Ω increased from 22 rev/s to 40 rev/s with an 85% increase. The change in the cell translational velocity is also presented in Figure 9b, where the average velocity of the cell increased from approximately 0.74 mm/s to 1.42 mm/s after the droplet was subjected to sheath flow (at *t* = 230 ms). This corresponds to a 90% increase in the average cell velocity (i.e., $I_{v}=90\%$).

**Figure 10:**
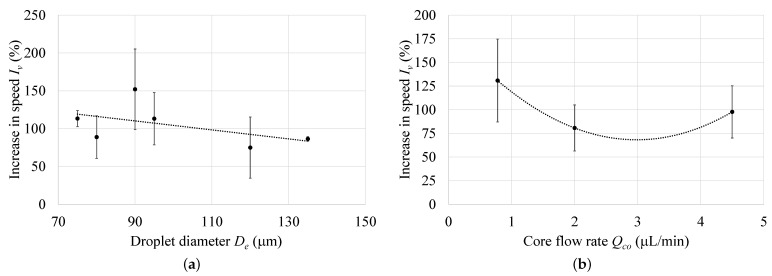
Increase in cell velocity ($I_{v}$) due to the sheath flow at different values of: (**a**) droplet diameter $D_{e}$, and (**b**) core flow rate $Q_{co}$.

The effect of parameters, including the droplet diameter $D_{e}$ and the core fluid flow rate $C_{co}$ on the increase in cell velocity ($I_{v}$) were also investigated. Figure 10a shows that the increase in $D_{e}$ led to a small reduction in $I_{v}$. Similarly, Figure 10b shows that $C_{co}$ had a limited effect on $I_{v}$. For all performed experiments, the velocity increase ($I_{v}$) when using a sheath flow rate ($Q_{s}$) of 1 μL/min was approximately 100%.

#### 5.5.2. Cell Dynamics—After Sheath

**Figure 11:**
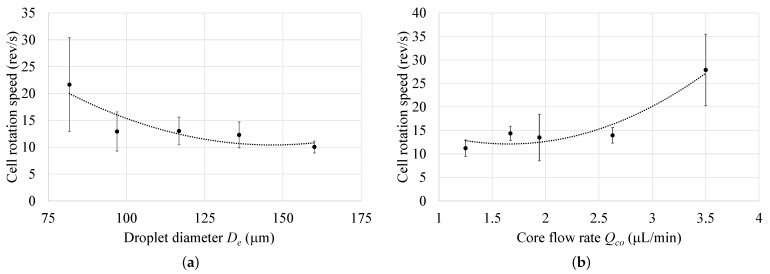
Cell rotational speed (Ω) after sheath flow at different values of: (**a**) droplet diameter $D_{e}$, and (**b**) core flow rate $Q_{co}$.

The effect of parameters, including the droplet diameter $D_{e}$ and the core fluid flow rate $C_{co}$ on the cell speed of rotation (Ω) after a sheath flow rate $Q_{s}=1$ μL/min, was investigated. Figure 11 reveals that Ω decreased as the droplet diameter increased. On the other hand, an increase in the core fluid flow rate $C_{co}$ led to an increase in Ω. Thus, to achieve a high cell rotation speed Ω, $C_{co}$ should be maximized, while keeping the dispersed-to-continuous flow rate ratio ($Q_{o}/Q_{w}$) small, which results in larger droplets [37].

## 6. Discussion and Conclusions

This paper studies the rotational flow fields inside water-in-oil emulsions flowing in Poiseuille flow conditions and how they affect the dynamics of encapsulated single cells. Numerical simulations were performed, which showed a velocity difference between the droplet and the external fluid. This disturbance causes the generation of a viscous force that opposes the interfacial tension, resulting in a rotational flow field inside the droplet. Simulation results showed that encapsulated cells spun in elliptical orbits, as was also confirmed experimentally. Results also revealed that the streamline along which a cell orbits depends on its initial position inside the droplet.

Tracking the cell dynamics, using a high-speed camera, can lead to the development of new label-free methods for the detection of rare cells based on their biomechanical properties. An encapsulated single cell being subjected to the droplet’s rotational flow fields responds by undergoing multiple orbits, spins, and deformations that depend on its physical properties. The encapsulated cell orbiting speed should be high to obtain sufficient data about the cell dynamics within field-of-view limitations. Sheath flow microchannels were therefore proposed to increase the relative velocity between the droplet and its surrounding fluid, thus strengthening the rotational flow field inside the droplet. This accelerates the rotational motion of the encapsulated cell.

Numerical simulations were again conducted for the system with sheath flow. Results revealed that the droplet diameter, droplet viscosity, and the sheath flow rate have the most significant effects on the acceleration of encapsulated cells. On the other hand, generated droplet diameters and core flow rates are the most significant parameters affecting cell velocity after subjection to sheath flow. The effect of sheath flow on the encapsulated cells dynamics was investigated experimentally using MCF-10A cells. The cell movement was tracked using image processing and feature recognition techniques. The ability of the proposed sheath flow technique to increase the translational velocity of encapsulated cells was evident for all performed experiments, where the cells speeds were almost doubled. Meanwhile, the effect of the droplet size or the core flow rate on the increase in speed was minimal. These results provide design guidelines for microfluidic chips, fluid selection, and flow rates for use in label-free biomechanical cell sorting.

## Figures and Tables

**Table 1:** Parameters with a potential effect on the rotational flow field and the average cell velocity $v_{c}$.

Parameter	Lower Value	Higher Value
Sheath flow rate $Q_{s}$ [µL/min]	0.5	2
Core flow rate $Q_{co}$ [µL/min]	0.5	5
Generated droplet diameter $D_{e}$ [µm]	70	190
Cell diameter $D_{c}$ [µm]	7	25
Continuous phase viscosity $\mu_{f}$ [mPa·s]	50	200
Droplet viscosity $\mu_{e}$ [mPa·s]	1	5

## Supplementary Materials

The following are available online at https://www.mdpi.com/article/10.3390/mi12070839/s1, Supplementary S1: More details about the numerical model.

## Abbreviations

The following abbreviations are used in this manuscript:

CFD	Computational Fluid Dynamics
PDMS	Polydimethylsiloxane

## References

[1] Suea-Ngam A., Howes P.D., Srisa-Art M., DeMello A.J. (2019). Droplet microfluidics: From proof-of-concept to real-world utility?. Chem. Commun..

[2] Ding Y., Howes P.D., deMello A.J. (2019). Recent advances in droplet microfluidics. Anal. Chem..

[3] Shang L., Cheng Y., Zhao Y. (2017). Emerging droplet microfluidics. Chem. Rev..

[4] Schneider T., Kreutz J., Chiu D.T. (2013). The potential impact of droplet microfluidics in biology. Anal. Chem..

[5] Ling S.D., Geng Y., Chen A., Du Y., Xu J. (2020). Enhanced single-cell encapsulation in microfluidic devices: From droplet generation to single-cell analysis. Biomicrofluidics.

[6] Chen Q., Lin J.M. (2019). Droplet-Based Microfluidics for Single-Cell Encapsulation and Analysis. Microfluidics for Single-Cell Analysis.

[7] Joensson H.N., Svahn H.A. (2012). Droplet microfluidics—A tool for single-cell analysis. Angew. Chem. Int. Ed..

[8] Mazutis L., Gilbert J., Ung W.L., Weitz D.A., Griffiths A.D., Heyman J.A. (2013). Single-cell analysis and sorting using droplet-based microfluidics. Nat. Protoc..

[9] Crippen S.M. (2013). Emulsion Biomechanics for Single Cells. Ph.D. Thesis.

[10] Eisenstein M. (2006). Cell sorting: Divide and conquer. Nature.

[11] Hulett H.R., Bonner W.A., Barrett J., Herzenberg L.A. (1969). Cell sorting: Automated separation of mammalian cells as a function of intracellular fluorescence. Science.

[12] Cho H., Kim J., Song H., Sohn K.Y., Jeon M., Han K.H. (2018). Microfluidic technologies for circulating tumor cell isolation. Analyst.

[13] Chen Y., Li P., Huang P.H., Xie Y., Mai J.D., Wang L., Nguyen N.T., Huang T.J. (2014). Rare cell isolation and analysis in microfluidics. Lab Chip.

[14] Shields IV C.W., Reyes C.D., López G.P. (2015). Microfluidic cell sorting: A review of the advances in the separation of cells from debulking to rare cell isolation. Lab Chip.

[15] Nasiri R., Shamloo A., Ahadian S., Amirifar L., Akbari J., Goudie M.J., Lee K., Ashammakhi N., Dokmeci M.R., Di Carlo D. (2020). Microfluidic-Based Approaches in Targeted Cell/Particle Separation Based on Physical Properties: Fundamentals and Applications. Small.

[16] Zheng S., Lin H., Liu J.Q., Balic M., Datar R., Cote R.J., Tai Y.C. (2007). Membrane microfilter device for selective capture, electrolysis and genomic analysis of human circulating tumor cells. J. Chromatogr. A.

[17] Pødenphant M., Ashley N., Koprowska K., Mir K.U., Zalkovskij M., Bilenberg B., Bodmer W., Kristensen A., Marie R. (2015). Separation of cancer cells from white blood cells by pinched flow fractionation. Lab Chip.

[18] Liu Z., Huang F., Du J., Shu W., Feng H., Xu X., Chen Y. (2013). Rapid isolation of cancer cells using microfluidic deterministic lateral displacement structure. Biomicrofluidics.

[19] Di Carlo D. (2009). Inertial microfluidics. Lab Chip.

[20] Yamada M., Seki M. (2005). Hydrodynamic filtration for on-chip particle concentration and classification utilizing microfluidics. Lab Chip.

[21] Alnaimat F., Dagher S., Mathew B., Hilal-Alnqbi A., Khashan S. (2018). Microfluidics based magnetophoresis: A review. Chem. Rec..

[22] Cheng I.F., Chang H.C., Hou D., Chang H.C. (2007). An integrated dielectrophoretic chip for continuous bioparticle filtering, focusing, sorting, trapping, and detecting. Biomicrofluidics.

[23] Petersson F., Åberg L., Swärd-Nilsson A.M., Laurell T. (2007). Free flow acoustophoresis: Microfluidic-based mode of particle and cell separation. Anal. Chem..

[24] Zhu Y. (2013). Micro segmented flow-functional elements and biotechnical applications. Front. Biosci..

[25] Vladisavljević G.T., Kobayashi I., Nakajima M. (2012). Production of uniform droplets using membrane, microchannel and microfluidic emulsification devices. Microfluidics Nanofluidics.

[26] Baroud C.N., Gallaire F., Dangla R. (2010). Dynamics of microfluidic droplets. Lab Chip.

[27] Spells K.E. (1952). A Study of Circulation Patterns within Liquid Drops moving through a Liquid. Proc. Phys. Soc. Sect. B.

[28] Fair R.B., Khlystov A., Tailor T.D., Ivanov V., Evans R.D. (2007). Chemical and biological applications of digital-microfluidic devices. IEEE Des. Test Comput..

[29] Kelly R. (2012). Advances in Microfluidics.

[30] Baret J.C. (2011). Surfactants in droplet-based microfluidics. Lab Chip.

[31] Taylor G.I. (1934). The Formation of Emulsions in Definable Fields of Flow. Proc. R. Soc. A Math. Phys. Eng. Sci..

[32] Taylor G.I. (1932). The Viscosity of a Fluid Containing Small Drops of Another Fluid. Proc. R. Soc. A Math. Phys. Eng. Sci..

[33] Galindo-Rosales F.J., Alves M.A., Oliveira M.S.N. (2013). Microdevices for extensional rheometry of low viscosity elastic liquids: A review. Microfluidics Nanofluidics.

[34] Lee J.S., Dylla-Spears R., Teclemariam N.P., Muller S.J. (2007). Microfluidic four-roll mill for all flow types. Appl. Phys. Lett..

[35] Wörner M. (2012). Numerical modeling of multiphase flows in microfluidics and micro process engineering: A review of methods and applications. Microfluidics Nanofluidics.

[36] Cristini V., Tan Y.C., Burns M.A., Johnson B.N., Brahmasandra S.N., Handique K., Webste (2004). Theory and numerical simulation of droplet dynamics in complex flows—A review. Lab Chip.

[37] Ibrahim A.M., Padovani J.I., Howe R.T., Anis Y.H. (2021). Modeling of Droplet Generation in a Microfluidic Flow-Focusing Junction for Droplet Size Control. Micromachines.

[38] González-Rodríguez R.M., Rial-Otero R., Cancho-Grande B., Simal-Gándara J. (2008). Occurrence of fungicide and insecticide residues in trade samples of leafy vegetables. Food Chem..

[39] Padovani J.I., Ibrahim A.M., Jeffrey S.S., Anis Y.H., Howe R.T. (2020). Electropermanent magnet-driven droplet size modulation for two-phase ferromicrofluidics. Microfluidics Nanofluidics.

[40] Padovani J.I., Jeffrey S.S., Howe R.T. (2016). Electropermanent magnet actuation for droplet ferromicrofluidics. Technology.

[41] Xia Y., Whitesides G.M. (1998). Soft lithography. Annu. Rev. Mater. Sci..

[42] Yuen H.K., Princen J.J.I., Kittler J. (1990). Comparative study of Hough transform methods for circle finding. Image Vis. Comput..

