# Supplementary material for: Encapsulated Cell Dynamics in Droplet Microfluidic Devices with Sheath Flow
Source: Micromachines (Basel). 2021 Jul 19;12(7):839. doi: 10.3390/mi12070839 (PMC8304737; doi:10.3390/mi12070839)
Supplement: Supplementary file 1 [file micromachines-12-00839-s001.zip › micromachines-1306403-supplementary.pdf]

1 Global Definitions

Global settings

COMSOL version	

Used products

COMSOL Multiphysics
Microfluidics Module

2 Component 1

Component settings

Unit system	SI

2.1 Definitions

2.1.1 Coordinate Systems

Boundary System 1

Coordinate system type	Boundary system
Tag	sys1

2.2 Geometry 1

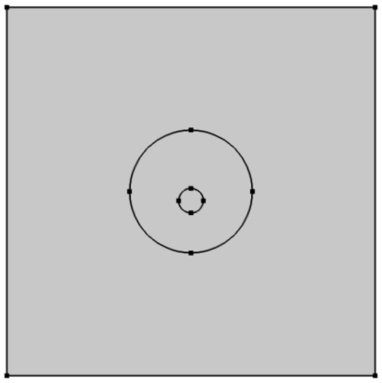

Geometry 1

Units

Length unit	µm
Angular unit	deg

Geometry statistics

Description	Value
Space dimension	2
Number of domains	3
Number of boundaries	12
Number of vertices	12

2.2.1 Rectangle 1 (r1)

Position

Description	Value
Position	{0, 0}
Base	Center

Size

Description	Value
Width	150
Height	150

2.2.2 Circle 1 (c1)

Position

Description	Value
Position	{0, 0}

Size and shape

Description	Value
Radius	25

2.2.3 Circle 2 (c2)

Position

Description	Value
Position	{0, -3.75}

Size and shape

Description	Value
Radius	5

2.3 Materials

2.3.1 Material 1

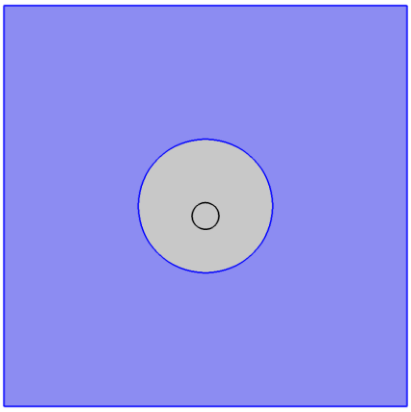

Material 1

Selection

Geometric entity level	Domain
Selection	Domain 1

Material parameters

Name	Value	Unit
Density	1[g/cm^3]	kg/m^3
Dynamic viscosity	150[mPa*s]	Pa*s

Basic Settings

Description	Value
Density	1[g/cm^3]
Dynamic viscosity	150[mPa*s]

2.3.2 Material 2

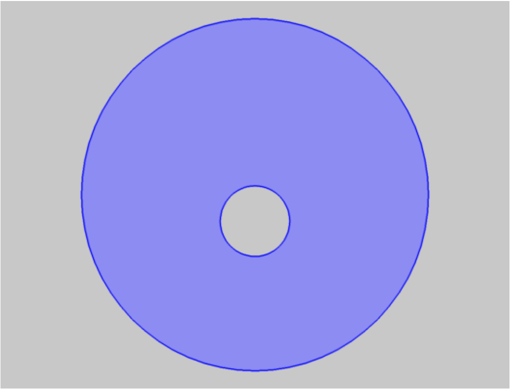

Material 2

Selection

Geometric entity level	Domain
Selection	Domain 2

Material parameters

Name	Value	Unit
Density	1[g/cm^3]	kg/m^3
Dynamic viscosity	1[mPa*s]	Pa*s

Basic Settings

Description	Value
Density	1[g/cm^3]
Dynamic viscosity	1[mPa*s]

2.3.3 Material 3

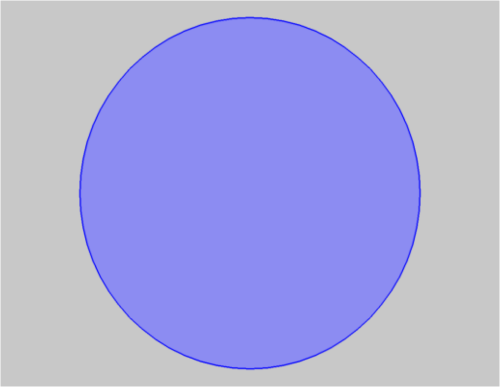

Material 3

Selection

Geometric entity level	Domain
Selection	Domain 3

Material parameters

Name	Value	Unit
Density	1[g/cm^3]	kg/m^3
Dynamic viscosity	100[mPa*s]	Pa*s

Basic Settings

Description	Value
Density	1[g/cm^3]
Dynamic viscosity	100[mPa*s]

2.4 Laminar Two-Phase Flow, Moving Mesh

Used products

COMSOL Multiphysics
Microfluidics Module

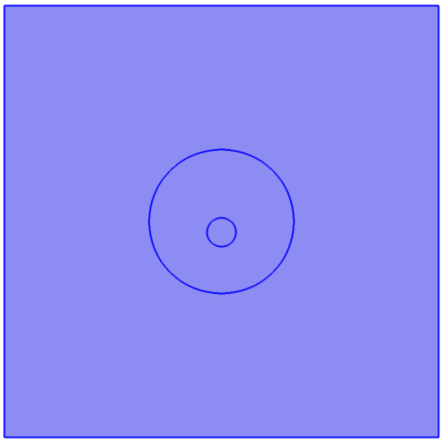

Laminar Two-Phase Flow, Moving Mesh

Selection

Geometric entity level	Domain
Selection	Domains 1–3

Equations

$$\rho\frac{\partial \mathbf{u}}{\partial t} + \rho(\mathbf{u}\cdot\nabla)\mathbf{u} =$$
$$\nabla\cdot\left[-p\mathbf{I} + \mu\left(\nabla\mathbf{u} + (\nabla\mathbf{u})^T\right)\right] + \mathbf{F}$$
$$\rho\nabla\cdot\mathbf{u} = 0$$

Settings

**Description**	**Value**
Discretization of fluids	P1 + P1
Value type when using splitting of complex variables	{Real, Real}
Material frame coordinates	{X, Y, Z}
Geometry shape order	1

2.4.1 Free Deformation 1

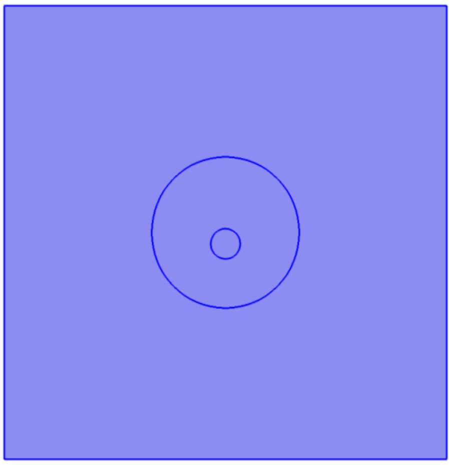

Free Deformation 1

Selection

Geometric entity level	Domain
Selection	Domains 1–3

Settings

Description	Value
Initial mesh displacement	{0, 0}

2.4.2 Prescribed Mesh Displacement 1

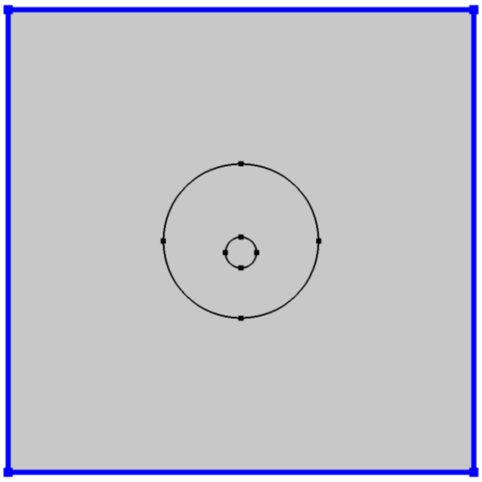

Prescribed Mesh Displacement 1

Selection

Geometric entity level	Boundary
Selection	Boundaries 1–4

Settings

Description	Value
Prescribed # displacement	{On, On}
Prescribed mesh displacement	{0, 0}
Use weak constraints	Off

2.4.3 Wall 1

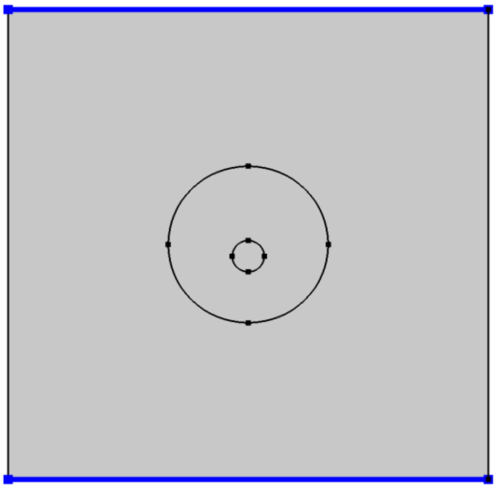

Wall 1

Selection

Geometric entity level	Boundary
Selection	Boundaries 2–3

Equations

$$\mathbf{u} = 0$$

Settings

Description	Value
Temperature	User defined
Temperature	293.15[K]
Electric field	User defined
Electric field	{0, 0, 0}
Boundary condition	No slip
Apply reaction terms on	Individual dependent variables
Use weak constraints	Off
Constraint method	Elemental

2.4.4 Fluid Properties 1

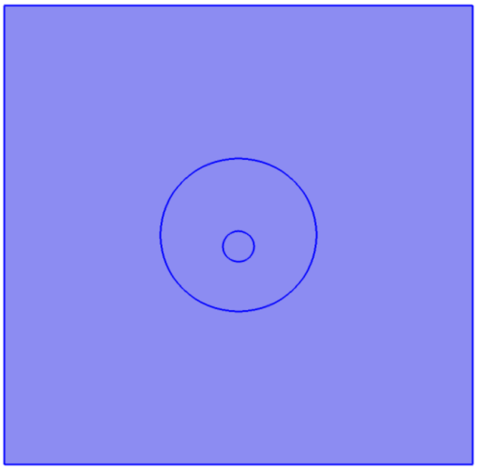

Fluid Properties 1

Selection

Geometric entity level	Domain
Selection	Domains 1–3

Equations

$$\rho\frac{\partial \mathbf{u}}{\partial t} + \rho(\mathbf{u}\cdot\nabla)\mathbf{u} =$$

$$\nabla\cdot\left[-p\mathbf{I} + \mu\left(\nabla\mathbf{u} + (\nabla\mathbf{u})^T\right)\right] + \mathbf{F}$$

$$\nabla\cdot\mathbf{u} = 0$$

Settings

Description	Value
Density	From material
Dynamic viscosity	From material
Reference length	1
Reference length scale	Automatic
Mixing length limit	Automatic

2.4.5 Initial Values 1

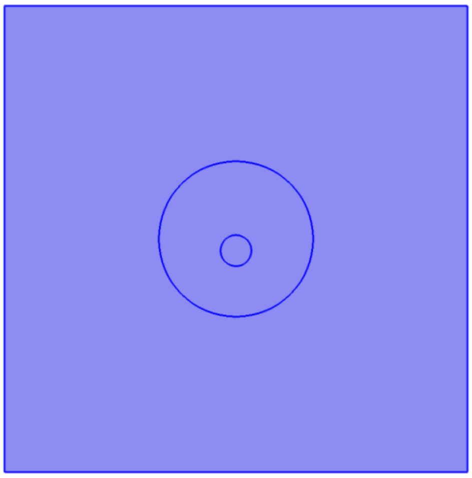

Initial Values 1

Selection

Geometric entity level	Domain
Selection	Domains 1–3

Settings

Description	Value
Velocity field	{0, 0, 0}
Pressure	0

2.4.6 Inlet 1

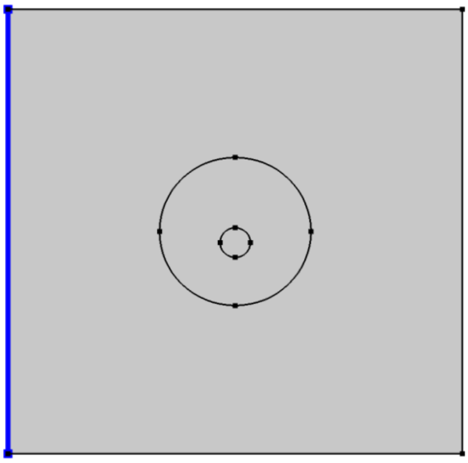

Inlet 1

Selection

Geometric entity level	Boundary
Selection	Boundary 1

Equations

$$L_{entr}\nabla_t \cdot \left[-p\mathbf{I} + \mu\left(\nabla_t \mathbf{u} + (\nabla_t \mathbf{u})^T\right)\right] = -p_{entr}\mathbf{n}$$

Settings

Description	Value
Boundary condition	Laminar inflow
Laminar inflow option	Flow rate
Flow rate	1[mm^3/min]
Entrance thickness	50[um]
Entrance length	1200[um]
Constrain endpoints to zero	Off
Standard pressure	1[atm]
Standard molar volume	0.0224136[m^3/mol]
Normal mass flow rate	1e-5[kg/s]
Mass flow type	Mass flow rate
Standard flow rate defined by	Standard density

2.4.7 Outlet 1

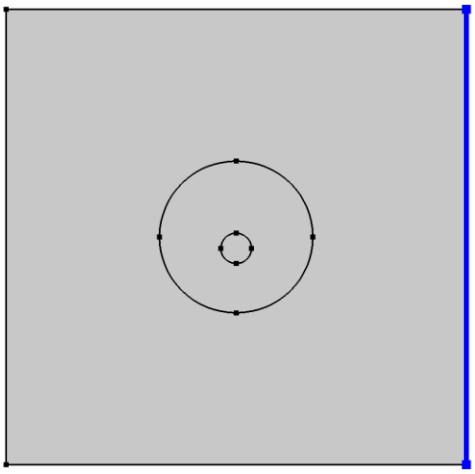

Outlet 1

Selection

Geometric entity level	Boundary
Selection	Boundary 4

Equations

$$\left[-p\mathbf{I} + \mu\left(\nabla \mathbf{u} + (\nabla \mathbf{u})^T\right)\right]\mathbf{n} = -\hat{p}_0\mathbf{n}$$

$$\hat{p}_0 \leq p_0$$

Settings

Description	Value
Boundary condition	Pressure
Pressure	0
Normal flow	Off
Suppress backflow	On
Apply reaction terms on	All physics (symmetric)
Use weak constraints	Off
Constraint method	Elemental

2.4.8 Fluid-Fluid Interface 1

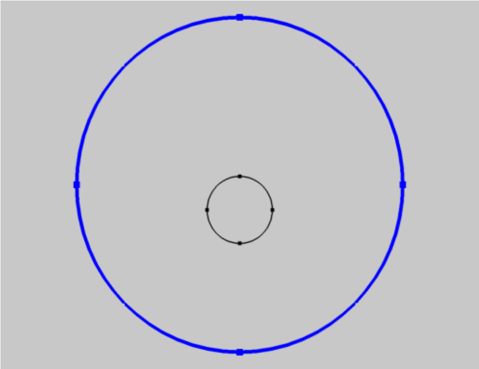

Fluid-Fluid Interface 1

Selection

Geometric entity level	Boundary
Selection	Boundaries 5–6, 9, 12

Equations

$$\mathbf{u}_1 = \mathbf{u}_2, \quad \mathbf{n}_1 \cdot \mathbf{T}_1 - \mathbf{n}_1 \cdot \mathbf{T}_2 = \sigma(\nabla_t \cdot \mathbf{n}_1)\mathbf{n}_1 - \nabla_t \sigma$$

$$\mathbf{u}_1 = \mathbf{u}_2 + M_f\left(\frac{1}{\rho_1} - \frac{1}{\rho_2}\right)\mathbf{n}_1$$

$$\mathbf{u}_{\mathrm{mesh}} = (\mathbf{u}_1 \cdot \mathbf{n}_1) - \frac{M_f}{\rho_1}\mathbf{n}_1$$

Settings

Description	Value
Mass flux	User defined
Mass flux	0
Surface tension coefficient	5[mN/m]
Surface tension coefficient	User defined

2.4.9 Fluid-Fluid Interface 2

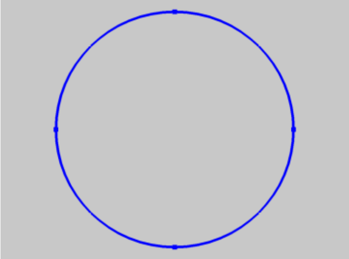

Fluid-Fluid Interface 2

Selection

Geometric entity level	Boundary
Selection	Boundaries 7–8, 10–11

Equations

$$\mathbf{u}_1 = \mathbf{u}_2, \quad \mathbf{n}_1 \cdot \mathbf{T}_1 - \mathbf{n}_1 \cdot \mathbf{T}_2 = \sigma(\nabla_t \cdot \mathbf{n}_1)\mathbf{n}_1 - \nabla_t \sigma$$

$$\mathbf{u}_1 = \mathbf{u}_2 + M_f\left(\frac{1}{\rho_1} - \frac{1}{\rho_2}\right)\mathbf{n}_1$$

$$\mathbf{u}_{mesh} = (\mathbf{u}_1 \cdot \mathbf{n}_1) - \frac{M_f}{\rho_1}\mathbf{n}_1$$

Settings

Description	Value
Mass flux	User defined
Mass flux	0
Surface tension coefficient	1[mN/m]
Surface tension coefficient	User defined

2.4.10 Volume Force 1

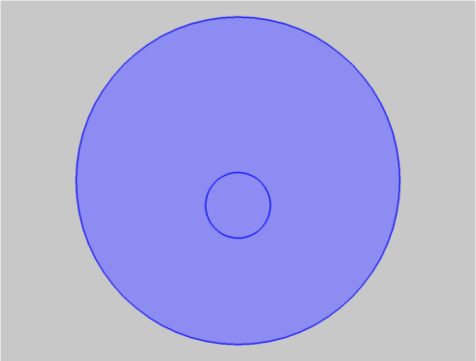

Volume Force 1

Selection

Geometric entity level	Domain
Selection	Domains 2–3

Equations

$$\rho\frac{\partial \mathbf{u}}{\partial t} + \rho(\mathbf{u}\cdot\nabla)\mathbf{u} =$$
$$\nabla\cdot\left[-p\mathbf{I} + \mu\left(\nabla\mathbf{u} + (\nabla\mathbf{u})^T\right)\right] + \mathbf{F}$$

Settings

Description	Value
Volume force	{-3.25[nN]/(4/3*pi*(25[um])^3), 0, 0}

2.5 Meshes

2.5.1 Mesh 1

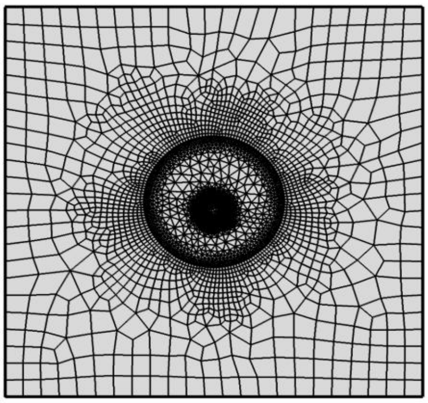

Mesh 1

Size (size)

Free Triangular 1 (ftri1)

Selection

Geometric entity level	Domain
Selection	Domains 2–3

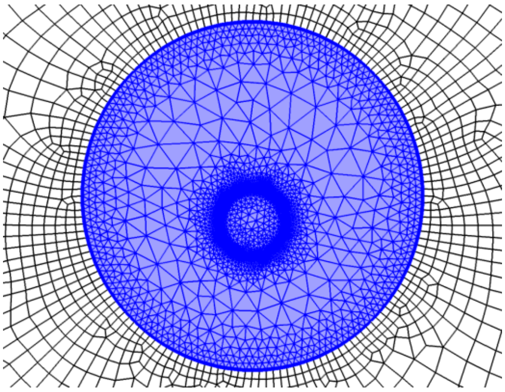

Free Triangular 1

Size 1 (size1)

Selection

Geometric entity level	Boundary
Selection	Boundaries 5–6, 9, 12

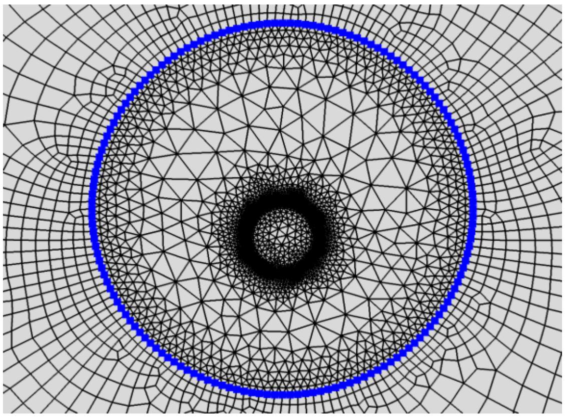

Size 1

Settings

Description	Value
Calibrate for	Fluid dynamics
Maximum element size	1.01
Minimum element size	0.003
Curvature factor	0.2
Maximum element growth rate	1.05
Predefined size	Extremely fine

Size 2 (size2)

Selection

Geometric entity level	Boundary
Selection	Boundaries 7–8, 10–11

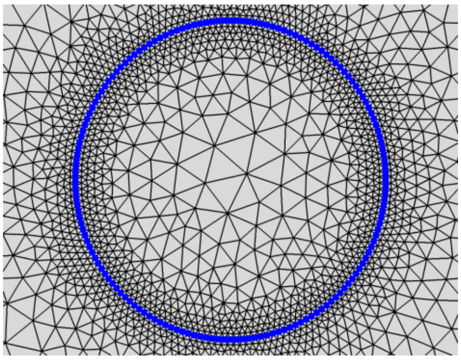

Size 2

Settings

Description	Value
Calibrate for	Fluid dynamics
Maximum element size	0.2
Minimum element size	0.003
Minimum element size	Off
Curvature factor	0.2
Curvature factor	Off
Resolution of narrow regions	Off
Maximum element growth rate	1.05
Maximum element growth rate	Off
Predefined size	Extremely fine
Custom element size	Custom

Free Quad 2 (fq2)

Selection

Geometric entity level	Domain
Selection	Domain 1

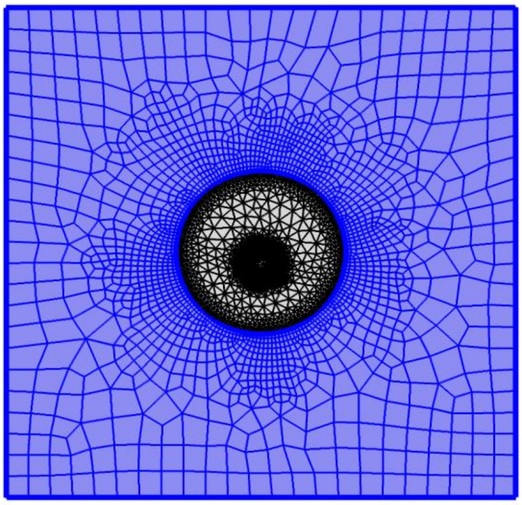

Free Quad 2

Size 1 (size1)

Selection

Geometric entity level	Boundary
Selection	Boundaries 1–4

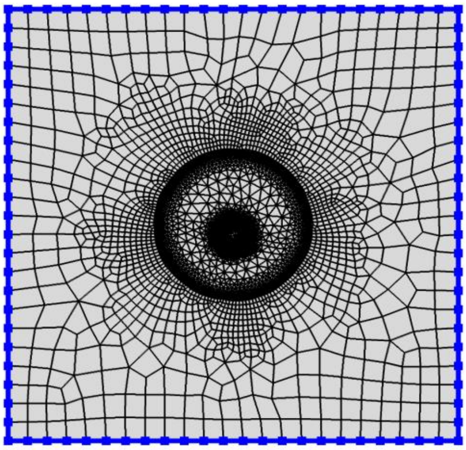

Size 1

Settings

Description	Value
Calibrate for	Fluid dynamics
Maximum element size	6.75
Minimum element size	0.3
Curvature factor	0.3
Maximum element growth rate	1.15

3 Study 1

Computation information

Computation time	19 min 23 s
CPU	Intel(R) Core(TM) i5-2410M CPU @ 2.30GHz, 2 cores
Operating system	Windows 7

3.1 Time Dependent

Study settings

Description	Value
Include geometric nonlinearity	Off

Times	Unit
range(0[ms],1[ms],600[ms])	s

Physics and variables selection

Physics interface	Discretization
Laminar Two-Phase Flow, Moving Mesh (tpfmm)	physics

Mesh selection

Geometry	Mesh
Geometry 1 (geom1)	mesh1

3.2 Solver Configurations

3.2.1 Solution 1

Compile Equations: Time Dependent (st1)

Study and step

Description	Value
Use study	Study 1
Use study step	Time Dependent

Dependent Variables 1 (v1)

General

Description	Value
Defined by study step	Time Dependent

Initial values of variables solved for

Description	Value
Solution	Remeshed Solution 1

Time-Dependent Solver 1 (t1)

Absolute tolerance

Description	Value
Global method	Unscaled

Time stepping

Description	Value
Maximum BDF order	2
Error estimation	Exclude algebraic

Results while solving

Description	Value
Plot	On
Plot group	Velocity (tpfmm)

Automatic Remeshing (arDef)

General

Description	Value
Remesh in geometry	Geometry 1

Condition for remeshing

Description	Value
Mesh quality expression	comp1.tpfmm.relVolMin
Stop when mesh quality is below	0.6

Fully Coupled 1 (fc1)

General

Description	Value
Linear solver	Direct 1

Method and termination

Description	Value
Jacobian update	On every iteration
Maximum number of iterations	8

Results while solving

Description	Value
Plot	On
Plot group	Velocity (tpfmm)

Direct 1 (d1)

General

Description	Value
Solver	PARDISO
Multithreaded forward and backward solve	Off
